# Nrf2 inhibits epithelial-mesenchymal transition by suppressing snail expression during pulmonary fibrosis

**DOI:** 10.1038/srep38646

**Published:** 2016-12-16

**Authors:** Wencheng Zhou, Xiaoting Mo, Wenhui Cui, Zhihui Zhang, Delin Li, Liucheng Li, Liang Xu, Hongwei Yao, Jian Gao

**Affiliations:** 1 School of Pharmacy, Anhui Medical University, Hefei, Anhui, 230032, China; 2 The Second Hospital of Dalian Medical University, Dalian, 116023, China; 3 The First Affiliated Hospital of Anhui Medical University, Hefei, 230022, China; 4 Anhui University of Chinese Medicine, Hefei, Anhui, 230038, China

## Abstract

Epithelial-mesenchymal transition (EMT) is a phenotype conversion that plays a critical role in the development of pulmonary fibrosis (PF). It is known that snail could regulate the progression of EMT. Nuclear factor erythroid 2 related factor 2 (Nrf2), a key regulator of antioxidant defense system, protects cells against oxidative stress. However, it is not known whether Nrf2 regulates snail thereby modulating the development of PF. Here, bleomycin (BLM) was intratracheally injected into both Nrf2-knockout (Nrf2^−/−^) and wild-type mice to compare the development of PF. Rat type II alveolar epithelial cells (RLE-6TN) were treated with a specific Nrf2 activator sulforaphane, or transfected with Nrf2 and snail siRNAs to determine their effects on transforming growth factor β1 (TGF-β1)-induced EMT. We found that BLM-induced EMT and lung fibrosis were more severe in Nrf2^−/−^ mice compared to wild-type mice. *In vitro*, sulforaphane treatment attenuated TGF-β1-induced EMT, accompanied by the down-regulation of snail. Inversely, silencing Nrf2 by siRNA enhanced TGF-β1-induced EMT along with increased expression of snail. Interestingly, when snail was silenced by siRNA, sulforaphane treatment was unable to reduce the progression of EMT in RLE-6TN cells. These findings suggest that Nrf2 attenuates EMT and fibrosis process by regulating the expression of snail in PF.

Pulmonary fibrosis (PF) is a devastating and disabling progressive lung disease, characterized by fibroblast proliferation and exaggerated accumulation of extracellular matrix (ECM), which finally leads to pulmonary architecture distortion and respiratory failure^1^. It is one of the most severe forms of lung diseases, with a median survival time of 2–3 years after diagnosis^2^. Unfortunately, nowadays there are no widely accepted treatments to protect against the progression of PF, which may be due to lacking of full understanding of pathogenetic mechanisms^3^.

Epithelial-mesenchymal transition (EMT) is a key event playing a critical role in the development of lung fibrotic disease^4^. Repetitive alveolar epithelial cell injury followed by the formation of fibroblastic foci could lead to an excessive deposition of ECM, which causes scarring and architectural distortion of the lung as well as irreversible loss of lung function^5^,^6^. EMT is a process in which epithelial cells gradually acquire mesenchymal features and enhance capacity for mesenchymal cross-talk. This is characterized by the loss of proteins associated with polarized epithelial phenotype such as E-cadherin and surfactant protein C (SPC), and the increase of mesenchymal markers such as vimentin and fibronectin^7^. In addition, the snail family of zinc-finger transcription factors (snail, slug, and smuc) have been identified as key EMT regulators, and are the ‘master switches’ critical for cell reprogramming^8^. Recent studies have shown that snail is a key transcription factor involved in the development of EMT, which could repress the expression of E-cadherin by direct binding to the E-box on its promoter, thereby promoting the progression of EMT^9^,^10^.

Nuclear factor E2-related factor 2 (Nrf2), belonging to the “cap ‘n’ collar” basic leucin zipper family, is a key orchestrator of cell responses to oxidative stress and protects against oxidant injury^11^,^12^. Under normal status, Nrf2 is anchored in the cytoplasm by Kelch-like ECH associated protein 1 (Keap1) which targets Nrf2 for ubiquitination and proteasomal degradation. Under conditions of electrophiles modification or oxidative stress, Nrf2-Keap1 interaction is disrupted and Nrf2 translocates to the nucleus, binds to the antioxidant-response element (ARE) of genes encoding the antioxidant and detoxifying enzymes^13^,^14^. Emerging evidence suggests that involvement of Nrf2-GSH signaling in transforming growth factor β1 (TGF-β1)-stimulated EMT in rat renal tubular cells, indicating that Nrf2 may be involved in regulating the development of EMT^15^. However, there are no direct evidences regarding the relationship between Nrf2 and EMT during lung fibrosis, and whether snail is involved in this process remains unknown. Here we hypothesized that Nrf2 protects against EMT by regulating snail during lung fibrosis. To test this hypothesis, Nrf2-deficient (Nrf2^−/−^) and wild-type (WT) mice were intratracheally instilled with bleomycin (BLM), followed by detecting the expression of EMT-related proteins and snail. Furthermore, in alveolar epithelial cell (AECs) RLE-6TN, we examined the levels of EMT markers and snail under TGF-β1 treatment in the presence of Nrf2 knockdown and activation.

## Results

### Relationship between Nrf2 and EMT in BLM-induced PF

**Figure 1:**
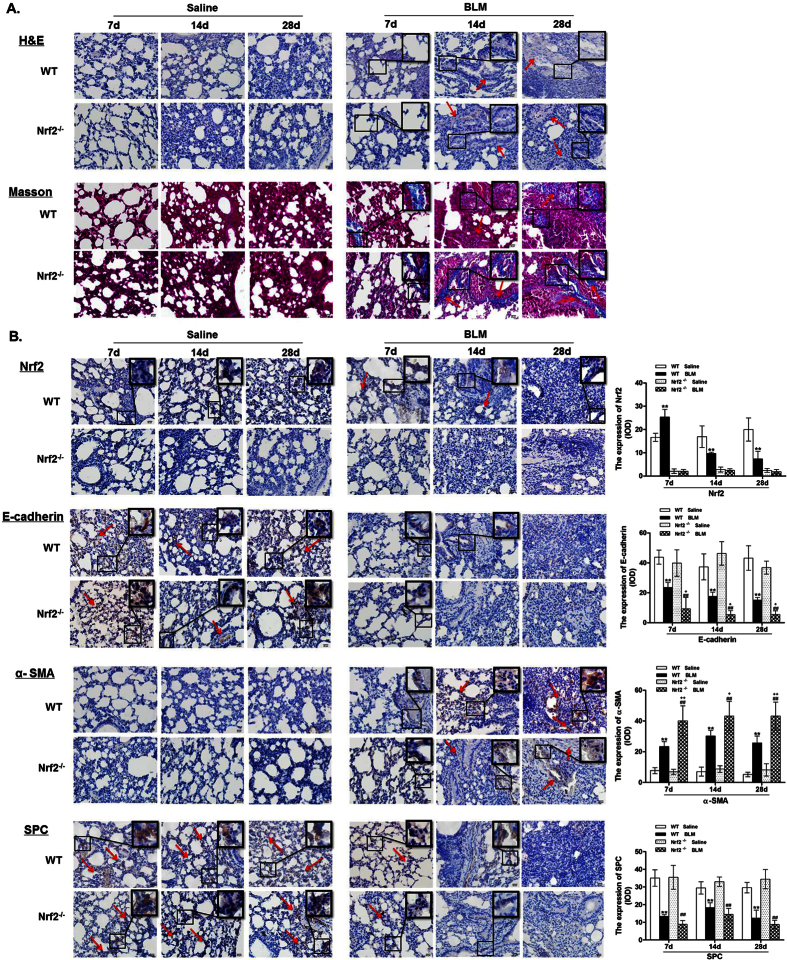
Relationship between Nrf2 and EMT in BLM-induced PF. Nrf2 knockout mice and wild-type mice were treated with BLM (4.5 mg/kg) or saline on days 7, 14 and 28. (**A**): Pulmonary tissue sections were stained with H&E and Masson’s trichrome to detect the histopathological structure and collagen accumulation respectively. Scale bars represent 20 μm. (**B**): Relative protein expression of Nrf2, E-cadherin, α-SMA and SPC was measured by IHC. Scale bars represent 20 μm. Histogram bars represent means ± SD (n = 4–6 per group). **P < 0.01 compared with WT saline group, ^##^P < 0.01 compared with Nrf2^−/−^ saline group, ^+^P < 0.05, ^++^P < 0.01 compared with WT BLM group.

**Figure 2:**
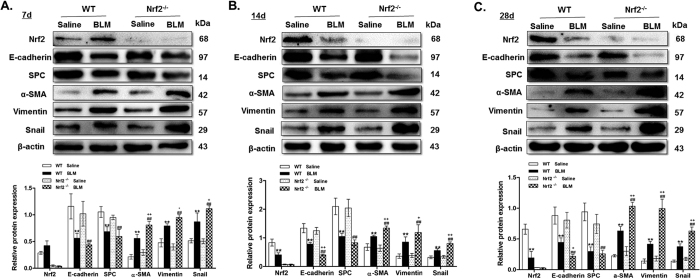
Relationship between Nrf2 and EMT in BLM-induced PF. Lung tissues were subjected to Western blot analysis for Nrf2, E-cadherin, SPC, α-SMA, vimentin and snail. The representative bands were obtained from different gels for repeated experiments. β-actin was used as an internal reference for relative quantification. Data represent the mean ± SD (n = 3–4 per group), **P < 0.01 compared with WT saline group; ^##^P < 0.01 compared with Nrf2^−/−^ saline group, ^+^P < 0.05, ^++^P < 0.01 compared with WT BLM group.

In order to investigate the relationship between Nrf2 and EMT in the pathogenesis of PF, we exposed Nrf2^−/−^ and WT mice to BLM or saline. Lung alveolar architecture damage and abnormal collagen deposition were observed in BLM-instilled WT mice, which were more severe in Nrf2^−/−^ mice. These results indicated that Nrf2^−/−^ mice were more susceptible to develop BLM-induced lung interstitial fibrosis compared to WT mice (Fig. 1A and Table 1). Next, we determined the changes in EMT-related proteins in lung tissues between Nrf2^−/−^ and WT mice by IHC and Western blot. It was found that on days 7, 14, and 28 post BLM instillation, the expression of epithelial cell marker E-cadherin was reduced in Nrf2^−/−^ mice as compared to WT mice, despite no significant changes in SPC abundance (Figs 1B and 2). In contrast, an increase in mesenchymal cell markers vimentin and α-smooth muscle actin (α-SMA) by BLM instillation was further augmented in Nrf2^−/−^ mice as compared to WT mice (Figs 1B and 2). These results clearly suggest that after BLM administration, EMT changes aggravate on days 7, 14, and 28, which is more severe in Nrf2^−/−^ mice as compared to WT mice.

### Pharmacological activation of Nrf2 attenuated TGF-β1-induced EMT in RLE-6TN cells

**Figure 3:**
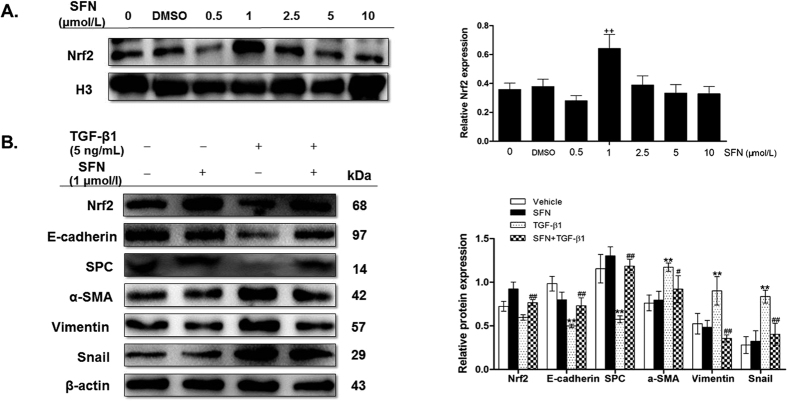
Activating Nrf2 attenuated TGF-β1-induced EMT in RLE-6TN cells. (**A**) Rats RLE-6TN cells were treated with SFN (0–10 μmol/L) for 24 h and the nuclear expression of Nrf2 was measured by Western blot. The representative bands were obtained from different gels for repeated experiments. Histone H3 was used as an internal reference for relative quantification. Data were expressed as mean ± SD (n = 3–4 per group), ^++^P < 0.01 compared with vehicle group. (**B**): Cells were cultured in the absence or presence of SFN (1 μmol/L) for 24 h, and then treated with TGF-β1 (5 ng/ml) for 24 h. Cell lysates were collected and the relative proteins were determined by Western blot. The representative bands were obtained from different gels for repeated experiments. β-actin was used as an internal reference for relative quantification. Data were expressed as mean ± SD (n = −4 per group). **P < 0.01 compared with vehicle group; ^#^P < 0.05, ^##^P < 0.01 compared with TGF-β1 group.

To determine whether activated Nrf2 attenuates EMT progression *in vitro*, RLE-6TN cells were incubated with different concentrations of sulforaphane (SFN), a known Nrf2 inducer, for 24 h. We found that 1 μmol/L of SFN was the optimum concentration, which induced a more evident nuclear expression of Nrf2 protein (Fig. 3A). Thus, we used 1 μmol/L of SFN in following experiments. As shown in Fig. 3B, compared to vehicle group, the expression of epithelial cell markers E-cadherin and SPC was decreased, while the expression of mesenchymal cell markers vimentin and α-SMA was increased in TGF-β1 group. Furthermore, we found that pre-treatment with SFN alleviated TGF-β1-induced EMT, with an up-regulation of epithelial cell markers E-cadherin and SPC but a down-regulation of mesenchymal cells markers α-SMA and vimentin. Additionally, SFN treatment reduced the expression of snail induced by TGF-β1 in RLE-6TN cells. These data suggest that activating Nrf2 attenuates TGF-β1-induced EMT markers in type II AECs RLE-6TN, which is associated with snail reduction.

### Silencing Nrf2 enhanced TGF-β1-induced EMT in RLE-6TN cells

**Figure 4:**
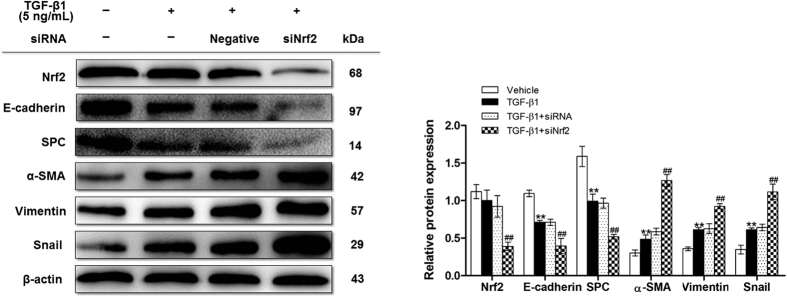
Silencing Nrf2 enhanced TGF-β1-induced EMT in RLE-6TN cells. Nrf2 siRNA were transfected in cells before stimulated with TGF-β1 for 24 h, then cell lysates were collected and the relative proteins were determined by Western blot. The representative bands were obtained from different gels for repeated experiments. β-actin was used as an internal reference for relative quantification. Data were expressed as mean ± SD (n = 3–4 per group), **P < 0.01 compared with vehicle group; ^##^P < 0.01 compared with TGF-β1 group.

To determine whether Nrf2 knockdown further enhances EMT, RLE-6TN cells were transfected with Nrf2 siRNA before TGF-β1 treatment, and EMT-related proteins were assessed by Western blot. As shown in Fig. 4, when Nrf2 was inhibited, TGF-β1-induced EMT was aggravated, which was accompanied by the up-regulation of mesenchymal cell markers α-SMA and vimentin as well as the down-regulation of epithelial markers E-cadherin and SPC. The level of snail was increased by Nrf2 siRNA transfection in RLE-6TN cells treated with TGF-β1 compared to scramble siRNA control. Taken together, our results support the initial hypothesis that Nrf2 protects against TGF-β1-mediated EMT changes and subsequent lung fibrosis.

### Nrf2 reduced the development of EMT via suppressing the expression of snail

**Figure 5:**
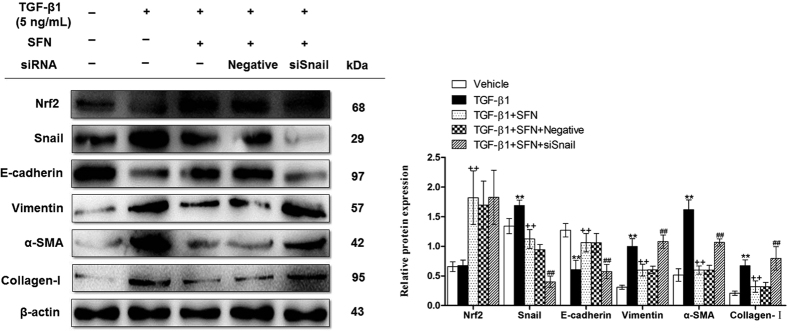
Nrf2 inhibited the development of EMT via suppressing the expression of snail. Snail siRNA were transfected in RLE-6TN cells, after 6 h incubation, the cells were treated with SFN for 24 h, followed by stimulation with TGF-β1 for 24 h. Cell lysates were collected and the relative proteins were determined by Western blot. The representative bands were obtained from different gels for repeated experiments. β-actin was used as an internal reference for relative quantification. Data were expressed as mean ± SD (n = 3–4 per group), **P < 0.01 compared with vehicle group; ^++^P < 0.01 compared with TGF-β1 group; ^##^P < 0.01 compared with TGF-β1+SFN group.

Although Nrf2 reduced TGF-β1-mediated EMT associated with snail reduction, it is not known whether snail reduction mediates Nrf2’s protection against EMT. To answer this question, RLE-6TN cells transfected with snail siRNA by Lipofectamine 2000 for 6 h were cultured with SFN for 24 h, following stimulated by TGF-β1 for 24 h. We found that when snail was silenced by siRNA, SFN treatment was unable to reduce the progression of EMT, which was reflected by the up-regulation of collagen-I, α-SMA and down-regulation of E-cadherin (Fig. 5). These findings demonstrate that Nrf2 regulates the progression of EMT by suppressing the expression of snail.

**Figure 6:**
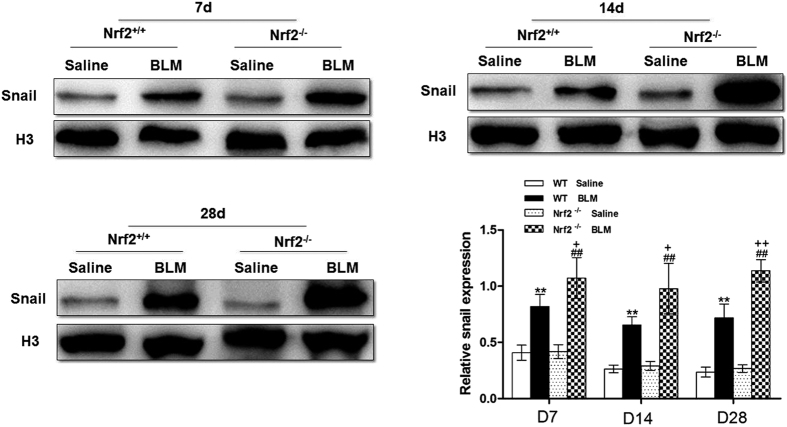
Nrf2 inhibited the development of EMT via suppressing the expression of snail. Nuclear snail protein level was assessed by Western blot in lung tissues. The representative bands were obtained from different gels for repeated experiments. Histone H3 was used as a loading control, and data were expressed as mean ± SD (n = 3–4 per group), **P < 0.01 compared with WT saline group; ^##^P < 0.01 compared with Nrf2^−/−^ saline group, ^+^P < 0.05, ^++^P < 0.01 compared with WT BLM group.

**Figure 7:**
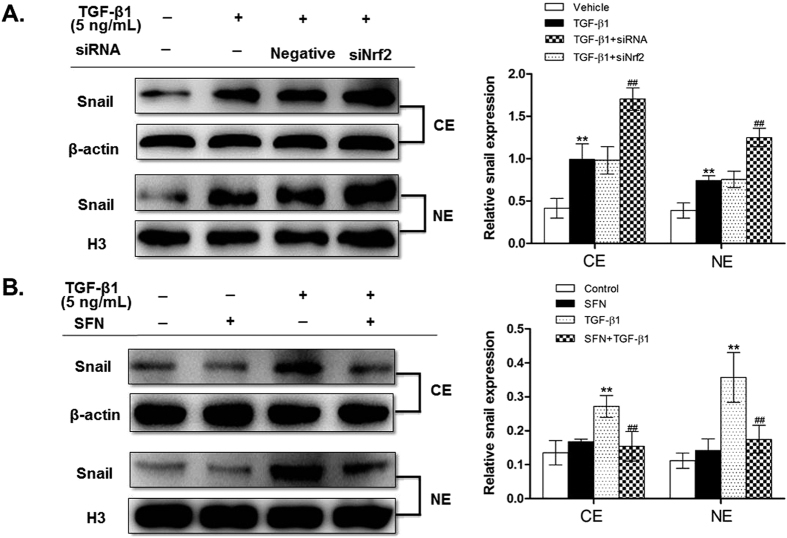
Nrf2 inhibited the development of EMT via suppressing the expression of snail. (**A**) The nuclear and cytoplasm expression of snail was assessed by Western blot after silencing Nrf2. (**B**) The nuclear and cytoplasm expression of snail was assessed by Western blot after activating Nrf2. The representative bands were obtained from different gels for repeated experiments. The densitometry values were normalized to β-actin or histone H3, respectively. Data were expressed as mean ± SD (n = 3–4 per group), **P < 0.01 compared with vehicle group; ^##^P < 0.01 compared with TGF-β1 group.

Snail is a transcription factor, which must translocate into nucleus to be functional^16^. Therefore, we determined nuclear and cytoplasmic levels of snail *in vivo* and *in vitro* by Western blot. We observed that on days 7, 14, and 28 post BLM instillation, a significant increase in nuclear and total snail expression was observed in lungs of Nrf2-deficient mice compared to WT mice (Figs 2 and 6). Similarly, Nrf2 knockdown by siRNA significantly increased total, cytoplasmic, and nuclear snail levels compared with vehicle group in RLE-6TN cells treated with TGF-β1 (Figs 4 and 7A). In contrast, SFN treatment significantly reduced total, cytoplasmic, and nuclear levels of snail in RLE-6TN cells treated with TGF-β1 stimulation (Figs 3 and 7B). Overall, these results illustrate that Nrf2 reduces nuclear translocation of snail protein during the progression of EMT.

## Discussion

PF is a chronic interstitial lung disease characterized by fibrosis of the lung parenchyma and loss of lung function. The etiology of PF is unknown, but aging, smoking, other environmental exposures, and infections have been reported as risk factors^17^. The pathological hallmark of PF is repetitive microscopic alveolar epithelial cell injury and dysregulated repair, fibroblast proliferation and excessive accumulation of ECM, which finally leads to lung architecture distortion and respiratory failure^5^. EMT has been implicated as the important mechanism of lung fibrogenesis through the generation of mesenchymal-type myofibroblasts from lung epithelial cells^18^. Meanwhile, the protective role of Nrf2 against fibrosis has been demonstrated in several studies, and Nrf2-deficient mice were susceptible to lung injury after exposure to bleomycin compared to WT mice^19^. This is corroborated by our findings that lung fibrotic responses to BLM were more severe and earlier in Nrf2^−/−^ mice compared to WT mice. Although Nrf2–antioxidant system was involved in TGF-β1-induced EMT during renal fibrosis, there are no direct evidences regarding the relationship between Nrf2 and EMT during PF^15^,^20^,^21^,^22^. In the present study, EMT markers were more obvious accompanied by the loss of epithelial marker E-cadherin as well as the increase of vimentin and α-SMA in Nrf2^−/−^ mice compared to WT mice. This is in agreement with our *in vitro* study using lung epithelial cells with genetic knockdown and pharmacological activator of Nrf2. These findings provide the evidence that Nrf2 protects against lung fibrosis via reducing EMT.

During the EMT, we noticed that the expression of epithelial cell marker SPC was not significantly reduced, despite BLM significantly decreased the expression of E-cadherin in Nrf2^−/−^ mice compared to WT mice. The reasons for these discrepancies are not known, which may be due to an Nrf2-specific effect. This needs further investigation.

It is known that EMT process can be mediated by activating a series of transcriptional regulators, such as snail, slug, and twist, and these transcription factors have been found to play crucial roles in promoting EMT^23^. Among them, snail is the most extensively studied, and snail protein is the first transcription factor discovered to repress CDH1 gene (encoding E-cadherin protein) transcription and induce EMT^24^. Increasing evidence shows that inducing the expression of snail promotes epithelial to mesenchymal transition and cells invasion^25^,^26^. A recent study reports that snail can be induced by TGF-β^27^. In agreement with our results, the cells were treated with TGF-β1 for 24 h, the expression of snail protein was augmented compared to vehicle group. Moreover, our previous studies also found that snail mediates the progression of EMT in RLE-6TN cells^28^. Here we explored the relationship between Nrf2 and snail *in vivo* and *in vitro*. We found that the level of snail was higher in Nrf2-deficient mice than that in WT mice when exposed to BLM, indicating that Nrf2 may regulate the expression of snail. Then, we proposed hypothesis that Nrf2 ameliorates the process of EMT through inhibiting the expression of snail. This is evidence by the following findings: 1) snail was increased in lungs of Nrf2^−/−^ mice exposed to BLM as compared to WT mice; 2) under TGF-β1-induced EMT, activing Nrf2 by SFN decreased whereas silencing Nrf2 by siRNA induced the expression of snail; 3) snail silence by siRNA attenuated the protective effects SFN on EMT. Nevertheless, whether the other transcription factors, such as slug and twist, are involved in the regulation of EMT and still need to be further explored. Future study using Nrf2 activators in snail-knockout mice will also detect whether Nrf2 modulates other transcription factors in addition to snail contributing to EMT.

Gene expression of snail transcription factor is modulated at the transcriptional level, while its activity is regulated by subcellular localization. Here, we purified and analyzed snail nuclear and cytoplasmic proteins *in vivo* and *in vitro*, and found that activing Nrf2 by SFN inhibited whereas Nrf2 knockdown increased both nuclear and cytoplasmic snail expression. This indicates that Nrf2 may regulate the transcription and translation process of snail directly or indirectly, which still needs to be further explored.

**Figure 8:**
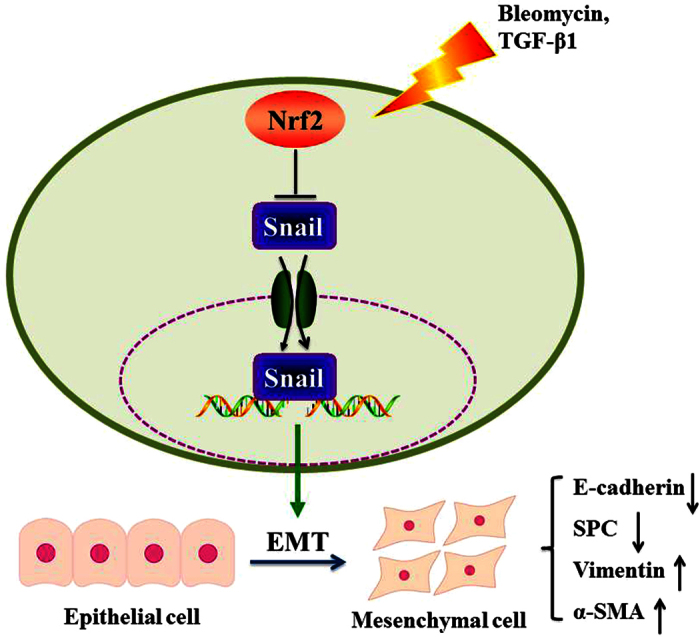
Nrf2 protected against the development of EMT by suppressing snail expression during pulmonary fibrosis.

In conclusion, Nrf2 protects against the development of EMT by suppressing snail expression during PF (Fig. 8). Therefore, Nrf2 may be a potential therapeutic target to prevent or attenuate EMT fibrosis process.

## Materials and Methods

### Ethics statement

All of the animal procedures involving mice, such as housing and care, and experimental protocols were approved by the Anhui Medical University Animal Care Committee and Use Committee. All procedures performed on the mice were conducted according to the guidelines from the National Institutes of Health.

### Animal model

Nrf2^−/−^ mice and their WT littermates were kindly provided by Drs. Peng Cao and Chunping Hu (Jiangsu Province Institute of Traditional Chinese Medicine, Nanjing, China), which were originally purchased from the Jackson Laboratory, USA (Order number: 3363093) and maintained in the SPF laboratory Experimental Animal Center of Anhui Medical University, Hefei, Anhui, China. Sixty Nrf2^−/−^ mice were randomly divided into saline group and BLM group (n = 30 per group), and sixty WT mice were also randomly assigned to two groups (n = 30 per group). All mice were housed in a specific pathogen-free environment with temperature (23 ± 2 °C), humidity (60 ± 10%) and light cycle (12:12 h light-dark), and were fed a purified diet and water ad libitum. The PF model was established through intratracheal instillation with 4.5 mg/kg BLM (Laiboten Pharmaceutical CO., LTD, Harbin, China), while the control group received the same volume of saline instead^19^. On days 7, 14 and 28 after BLM instillation, these mice were anaesthetized with 10% chloral hydrate intraperitoneally, and lung tissues were collected for further analysis.

### Western blot analysis

Lung tissues or cells were lysed with radio immunoprecipitation assay buffer (RIPA; P0013C, Beyotime Institute of Biotechnology, China) including 1 mM proteinase inhibitor phenylmethylsulfonyl-fluoride (PMSF; Amresco 0754, Biosharp, USA). The supernatant was collected in eppendorf tubes through centrifugation (12,000 r/min, 10 min at 4 °C), mixed with loading buffer (4:1), heated in boiling water for 10 min, and stored in −20 °C. Protein samples were subjected to 10%–12% sodium dodecyl sulfate polyacrylaminde gel electrophoresis (SDS-PAGE) and transferred to polyvinyl difluoride (PVDF) membranes (IPVH00010; Millipore, USA). The membranes were blocked with 5% non-fat milk (Guangming, China) for 2 h, followed by overnight incubation with primary antibody at 4 °C, including anti-E-cadherin (ab76055, abcam), anti-α-SMA (ab5694, abcam), anti-vimentin (ab92547, abcam), anti-SPC (sc-7705, abcam), anti-snail (ab180714, abcam), anti-Nrf2 (ab31163, abcam), and anti-β-actin (ab52614, abcam), all of which were purchased from USA. On the next day, the membranes were incubated with appropriate secondary antibodies (ZSGB-BIO, Beijing, China) for 1 h at room temperature after washing 3 times (10 minutes each time). Finally, the signals were visualized using the enhanced chemiluminescence reagent (ECL; Thermo Scientific, Rockford, USA), and β-actin was used as an internal reference for relative quantification.

### Preparation of nuclear and cytoplasmic extracts

Nuclear proteins were purified with nuclear and cytoplasmic protein extraction kit (KeyGEN BioTECH, Nanjing, China) according to the manufacturer’s instruction^29^. Briefly, lung tissues or cells were mechanically homogenized in ice-cold buffer A (10 mmol/L Hepes (pH 7.5), 10 mmol/L KCl, 1.5 mmol/L MgCl_2_, 0.5 mmol/L DTT, 1 mmol/L NaF, 1 mmol/L glycerol phosphate, and 1 protease inhibitor cocktail.) for 10 min, and mixed with buffer B (10 mmol/L Hepes (pH 7.5), 10 mmol/L KCl, 1.5 mmol/L MgCl_2_, 0.5 mmol/L DTT, 1 mmol/L NaF, 1 mmol/L glycerol phosphate, 1 protease inhibitor cocktail and 0.15% of Nonident P-40) for 1 min, followed by centrifugation at 16,000 rpm for 10 min, the supernatant was collected as the cytoplasmic protein and stored at −80 °C until use. Then the pellet was resuspended in 100 μl of buffer C (20 mmol/L Hepes (pH 7.5), 420 mmol/L NaCl, 1.5 mmol/L MgCl_2_, 0.5 mmo/L DTT, 1 mmol/L NaF, 1 mmol/L glycerol phosphate, and 1 protease inhibitor cocktail) and placed on the rotating rocker in the cold room for 30 min, and further centrifuged for 30 min at 16,000 rpm 4 °C. Finally, the supernatant was transferred into a 1.5 ml eppendorf tube and collected as nuclear proteins and stored at −80 °C until use. Proteins in the nuclear extract were quantified by BCA protein assay.

### Histopathologic assessment and immunohistochemistry

Hematoxylin and eosin (H&E), Masson’s trichrome and immunohistochemistry (IHC) staining were performed as previously described^30^,^31^,^32^. Briefly, lung tissues were fixed using 10% formaldehyde solutions for 24 h, followed by paraffin-embedding, and the paraffin blocks were cut at 5 μm using microtome. Then deparaffinized tissue slices were stained with H&E and Masson’s trichrome for histological examination. The expression of E-cadherin, SPC, α-SMA and Nrf2 was investigated by using immunohistochemistry in lung tissues. Briefly, the deparaffinized and rehydrated lung sections were incubated with 3% H_2_O_2_ in methanol for 30 min to block endogenous peroxidase activity. Nonspecific binding of antibodies to the tissue sections was blocked with 1.5% normal goat serum in PBS with 0.5% bovine serum albumin. Subsequently, lung tissue sections were incubated with primary anti-E-cadherin, anti-SPC, anti-α-SMA and anti-Nrf2 antibodies at corresponding 1:250, 1:500, 1:200 and 1:100 dilution at 37 °C for 30 min, and then kept at 4 °C overnight. After being washed, the slides were incubated with secondary antibody for 10 min at 37 °C. Next, the sections were incubated with streptavidin-biotin-peroxidase complex for 10 min, and diaminobenzidine (DAB) was added as a visualizing agent. The counterstaining with hematoxylin was then performed before examination under a light microscope. The images were captured at 200x magnification on a light microscope (Olympus, Tokyo, Japan).

The pathologic scores of alveolitis and fibrosis are based on the research of Szapiel, *et al*.^33^. H&E staining was used to evaluate pathological changes in lung tissues. The alveolitis classification was as follows: no alveolitis (−), mild alveolitis (+; affected area <20%), moderate alveolitis (++; affected area ~20–50%), and severe alveolitis (+++; affected area >50%). Masson’s trichrome was used to evaluate pulmonary fibrosis. The classification of pulmonary fibrosis was as follows: no pulmonary fibrosis (−), light degree of pulmonary fibrosis (+; lesion range <20% in the whole lung), moderate pulmonary fibrosis (++; lesion range ~20–50% in the whole lung), and severe pulmonary fibrosis (+++; lesion range >50% in the whole lung, accompanied by alveolar fusion and lung parenchyma structural disorder). The degree of PF was recorded as 0, 1, 2 and 3 points, which was correlated with −, +, ++ and +++, respectively.

### Cell line and culture

Rat type II AECs (RLE-6TN) were purchased from ATCC (Manassas, USA), and grown in 1640 medium (Gibco, USA) supplemented with 10% fetal bovine serum (FBS; Gibco, USA), and the cells were incubated at 37 °C in a humidified atmosphere with 5% CO_2_. In some experiments, the cells were incubated with recombinant human TGF-β1 (100–21 C, PeproTech) or SFN (1 μmol/L, Sigma S6317)^20^,^28^. Then, cell lysates were harvested for Western blot analysis.

### Transfection

The Nrf2 small interference RNA (siRNA), snail siRNA, and negative control siRNA were designed and synthesized by GenePharma (Shanghai, China). The sequences were shown as follows: Nrf2, the forward primer was 5′-GAGGAUGGGAAACCUUACUTT-3′ and the reverse primer was 5′-AUAUUUGCAGUUGAAGGCCTT-3′. Snail, the forward primer was 5′-GGCCUUCAACUGCAAAUAUTT-3′ and the reverse primer was 5′-AUAUUUGCAGUUGAAGGCCTT-3′. These siRNAs were transfected into RLE-6TN cells by use of Lipofectamine 2000 (Invitrogen, USA) as per the manufacturer’s instructions.

### Statistical analysis

Values were presented as mean ± standard deviation (SD). Between-group differences were assessed by the Student’s t-test, and a one-way analysis of variance (ANOVA) was used to analyze three or more groups. The scores of alveolitis and fibrosis were evaluated. All analyses were performed by SPSS 13.0 software, and values of p < 0.05 were considered statistically significant.

## Additional Information

**How to cite this article**: Zhou, W. *et al*. Nrf2 inhibits epithelial-mesenchymal transition by suppressing snail expression during pulmonary fibrosis. *Sci. Rep.* **6**, 38646; doi: 10.1038/srep38646 (2016).

**Publisher's note:** Springer Nature remains neutral with regard to jurisdictional claims in published maps and institutional affiliations.

## Figures and Tables

**Table 1:** Pathologic scores of alveolitis and fibrosis.

Groups	Alveolitis			Fibrosis		
	7 days	14 days	28 days	7 days	14 days	28 days
WT saline	0.17 ± 0.41	0.17 ± 0.41	0.17 ± 0.41	0.00 ± 0.00	0.00 ± 0.00	0.00 ± 0.00
WT BLM	1.50 ± 0.55**	1.67 ± 0.52**	2.17 ± 0.41**	1.50 ± 0.55**	1.33 ± 0.52**	2.00 ± 0.89**
Nrf2^−/−^ saline	0.17 ± 0.41	0.17 ± 0.41	0.17 ± 0.41	0.00 ± 0.00	0.00 ± 0.00	0.00 ± 0.00
Nrf2^−/−^ BLM	2.50 ± ± 0.55^##^	2.50 ± 0.84^##^	2.67 ± 0.52^#^	2.17 ± 0.75^#^	2.67 ± 0.52^##^	2.83 ± 0.41^##^

Data are means ± SD (n = 6).

**p < 0.01 compared with WT saline.

^#^p < 0.05, ^##^p < 0.01 compared with WT BLM.
